# Exploratory study of sea buckthorn enhancing QiangGuYin efficacy by inhibiting CKIP-1 and Notum activating the Wnt/β-catenin signaling pathway and analysis of active ingredients by molecular docking

**DOI:** 10.3389/fphar.2022.994995

**Published:** 2022-10-11

**Authors:** Yi-Feng Yuan, Shen Wang, Hang Zhou, Bin-Bin Tang, Yang Liu, Hai Huang, Cai-Jian He, Tian-Peng Chen, Mou-Hao Fang, Bo-Cheng Liang, Ying-De-Long Mao, Feng-Qin Qie, Kang Liu, Xiao-Lin Shi

**Affiliations:** ^1^ The Second School of Clinical Medicine, Zhejiang Chinese Medical University, Hangzhou, China; ^2^ The Second Affiliated Hospital of Zhejiang Chinese Medical University (Xinhua Hospital of Zhejiang Province), Hangzhou, China; ^3^ Yushi Health Research Institute, Tokyo, Japan

**Keywords:** sea buckthorn, postmenopausal osteoporosis, Notum, CKIP-1, Wnt/β-catenin signaling pathway, molecular docking, QiangGuYin

## Abstract

**Background:** Sea buckthorn (SBT) is a traditional Chinese medicine (TCM), rich in calcium, phosphorus, and vitamins, which can potentially prevent and treat osteoporosis. However, no research has been conducted to confirm these hypotheses. QiangGuYin (QGY) is a TCM compound used to treat osteoporosis. There is a need to investigate whether SBT enhances QGY efficacy.

**Objectives:** The aim of this study was to explore whether SBT enhances QGY efficacy by inhibiting CKIP-1 and Notum expression through the Wnt/β-catenin pathway. The study also aimed to explore the active components of SBT.

**Methods:** Experimental animals were divided into control, model, QGY, SBT, SBT + *Eucommia ulmoides* (EU), and SBT + QGY groups. After treatment, bone morphometric parameters, such as estrogen, PINP, and S-CTX levels, and Notum, CKIP-1, and β-catenin expression were examined. Screening of SBT active components was conducted by molecular docking to obtain small molecules that bind Notum and CKIP-1.

**Results:** The results showed that all the drug groups could elevate the estrogen, PINP, and S-CTX levels, improve femoral bone morphometric parameters, inhibit Notum and CKIP-1 expression, and promote β-catenin expression. The effect of SBT + EU and SBT + QGY was superior to the others. Molecular docking identified that SBT contains seven small molecules (folic acid, rhein, quercetin, kaempferol, mandenol, isorhamnetin, and ent-epicatechin) with potential effects on CKIP-1 and Notum.

**Conclusion:** SBT improves bone morphometric performance in PMOP rats by inhibiting CKIP-1 and Notum expression, increasing estrogen levels, and activating the Wnt/β-catenin signaling pathway. Furthermore, SBT enhances the properties of QGY. Folic acid, rhein, quercetin, kaempferol, mandenol, isorhamnetin, and ent-epicatechin are the most likely active ingredients of SBT. These results provide insight into the pharmacological mechanisms of SBT in treating osteoporosis.

## Introduction

Postmenopausal osteoporosis (PMOP) is the most common type of osteoporosis in women within 5–10 years of menopause and is characterized by estrogen deficiency, decreased bone mineral density, and impaired bone quality. PMOP is similar to primary osteoporosis regarding the imbalance in bone metabolism and a bias toward heightened bone resorption activity. Unique PMOP characteristics include a high bone turnover rate, that is, a high turnover type of bone metabolism. After the completion of each bone turnover, the residual resorption lacunae and unmineralized new bone exponentially increase in the high turnover state, and the corresponding indexes of bone metabolism also rise. QiangGuYin (QGY) is a compound developed by Prof. Xiao-lin Shi to treat PMOP and is patented in China (Patent No. ZL200610053058.8). Several clinical studies have confirmed the efficacy of QGY in treating PMOP. QGY is highly effective in elevating bone mineral density, reducing the risk of falling, and alleviating osteoporotic pain (Shi, Liu, and Li, 2007; Shi et al., 2008, 2017; Xiao et al., 2019; Li et al., 2021; Mao et al., 2021; Hui et al., 2022). Herein, we evaluated the clinical efficacy of QGY for PMOP and hope to further enhance its efficacy.

Sea buckthorn (SBT) is a spiny nitrogen-fixing deciduous shrub cultivated worldwide for its nutritional and medicinal values. Fruits, seeds, and other parts of SBT are rich in vitamins A, B1, B12, C, E, K, and P, flavonoids, lycopene, carotenoids, and plant sterols. SBT is therapeutically important because it is rich in potent antioxidant substances. These molecules possess antioxidant, anticoagulant, anticancer, wound healing, anti-inflammatory, and radioprotective properties (Suryakumar and Gupta, 2011; Patel et al., 2012). Among them, antioxidant and anti-inflammatory effects inhibit or alleviate PMOP. Studies showed that SBT fatty acids significantly increase serum estrogen, insulin-like growth factor (IGF), transforming growth factor (TGF) levels, trabecular number, cortical bone thickness, and maximum bone stress resistance and improve the bone density in old female rats (Liu, Yuan, Guo, Wei, Zhao, Chen, et al., 2006; Liu, Yuan, Guo, Wei, Zhao, Kang, et al., 2006). Therefore, SBT has considerable potential in treating osteoporosis, and its efficacy, mechanism of action, and active ingredients should be explored. At the same time, we explored whether SBT can enhance the efficacy of QGY.

Our previous study found that CKIP-1 regulates bone metabolism (Liu et al., 2018; Yang et al., 2018) and that QGY acts as an anti-osteoporosis agent by inhibiting CKIP-1 and Akt binding in osteoblasts, activating the Akt/mTOR signaling pathway, inhibiting cellular autophagy, promoting osteogenic differentiation, and promoting osteoporosis (Yuan et al., 2022). QGY also influences osteoblast differentiation through the Wnt/β-catenin pathway by affecting the expression of related miRNAs in the exostome of osteoclasts (Tang et al., 2020). Notum is a carboxyl esterase that catalyzes the deacetylation and inactivation of Wnts by inhibiting the Wnt signaling pathway (Kakugawa et al., 2015; Zhang et al., 2015). Therefore, animal experiments were used in this study to investigate the efficacy of SBT in treating PMOP and augmenting the efficacy of QGY. The mechanism through which SBT exerts its effect was also explored. The main active components in SBT that mediate its effects were identified by molecular docking.

## Materials and methods

### Ethics statement

The protocol for this study was approved by the Laboratory Animal Management and Ethics Committee of Zhejiang Chinese Medical University (Approval Number: IACUC-20220613-01). All animal experiments were conducted in accordance with the Guidelines for the Care and Use of NIH Laboratory Animals (NIH Publication No. 80–23, revised in 1978).

### Experimental animals

Mature female Sprague–Dawley (SD) rats (n = 34, 6 ∼ 8-week-old, 200 ± 20 g in weight) were purchased from the Experimental Animal Center of Hangzhou Medical College (production license no. SCXK 2019–0002, Hangzhou, China) and were provided with enough food and water. The rats were reared in a specific pathogen-free room at 20–25°C, with a relative humidity of 60 ± 5%, and 12 h light/dark cycle.

### Preparation of medicines

SBT, *Eucommia ulmoides* (EU), and QGY were purchased from the Pharmaceutical Preparation Center of the Second Affiliated Hospital of Zhejiang Chinese Medical University. Each preparation was a 22.2 ml formulation containing a different combination of drugs. The SBT group is composed of 20 g of SBT, and the SBT + EU group comprised 20 g of SBT and 10 g of EU. The QGY group comprised 245 g of crude drugs, including 30 g of Astragali Radix, 30 g of Dipsaci Radix, 25 g of Lonicerae Japonicae Caulis, 25 g of Spatholobi Caulis, 20 g of Drynariae Rhizoma, 20 g of Chuanxiong Rhizoma, 20 g of Cervi Cornu Degelatinatum, 20 g of Vespae Nidus, 15 g of *Eucommia ulmoides*, 15 g of Gentianae Macrophyllae Radix, 15 g of Saposhnikoviae Radix*,* and 10 g of Cinnamomi Cortex. The SBT + QGY group comprised 245 g of the crude drugs and 20 g of SBT.

### Grouping and treatment

A total of 34 rats were randomly divided into six groups: 1) control group (n = 4); 2) model group (n = 6); 3) SBT group (n = 6); 4) SBT + EU group (n = 6); 5) SBT + QGY group (n = 6); and 6) QGY group (n = 6). Except for the control group, all the other groups underwent bilateral oophorectomy. The rats were acclimatized for 10 weeks before receiving the treatments.

The corresponding drug was administered by gavage at a dose of 10 ml/kg twice daily. The rats in the normal control group and model group received physiological saline. The treatments lasted 6 weeks, after which the right femur bones of mice in each group were extracted for a micro-CT scan.

### Histomorphometric analysis

The rats were scanned from the hip region to the limb using an animal X-ray imaging system (Mikasa X-ray HF100HA, Mikasa, Japan) to measure the hip bone width and obtain macroscopic imaging of the lower limbs. Bone tissue analysis was conducted using a micro-CT imaging system (Micro-CT μCT100, SCANCO Medical, Sweden). The right femur sample was scanned at 14.8 μm resolution. Trabecular bone regions of interest were selected for three-dimensional reconstruction. The parameters measured using the reconstructed images include bone mineral density (BMD), connectivity density (Conn.D.), bone mineral content (BMC), trabecular thickness (Tb.Th), trabecular number (Tb.N), bone volume fraction (BV/TV), and trabecular separation (Tb.Sp), which reflected the internal microarchitecture of the femur.

### Western blot analysis

To evaluate the effect of each drug on the expression of Notum and CKIP-1 and the regulation of the Wnt/β-catenin signaling pathway, the bone marrow of the right tibia was analyzed by Western blotting. The antibodies used include Notum (Notum rabbit antibody, dilution 1:1,000, Proteintech, Chicago, United States), CKIP-1 (CKIP-1 rabbit antibody, dilution 1:1,000, Proteintech, Chicago, United States), and β-catenin (β-catenin rabbit antibody, dilution 1:1,000, Proteintech, Chicago, United States). The mean normalized protein expression ±S.D. was calculated from independent experiments. GAPDH (GAPDH polyclonal antibody, dilution 1:10,000, Proteintech, Chicago, United States) was used as the internal control.

### ELISA analysis

The estrogen level, bone turnover markers, C-terminal cross-linking telopeptide of type I collagen (S-CTX), and pro-collagen type I N-terminal propeptide (PINP) in the orbital blood of rats were measured using enzyme-linked immunosorbent assay (ELISA) analysis.

### Molecular docking of sea buckthorn

#### Platform and software

Computational simulation and data processing were conducted using the Microsoft Windows 10 Professional operating system, whereas BIOVIA Discovery Studio 2019 (DS) was used for molecular docking research. Default settings were used except where specified.

### Receptor protein preparation

Notum (PDB ID: 4UZQ) and CKIP-1(PDB ID: 3AA1) protein crystal models were derived from human-derived protein crystallization in the PDB database (http://www.rcsb.org). The amino acid sequences and protein space models of 4UZQ and 3AA1 were imported using the BLAST search module in DS, followed by both protein preparation and structure processing of protein crystallization. Water molecules and alternate conformations were removed, atomic names were standardized, and missing residues were restored through complementation to obtain a prototype of the actual structure.

### Determination of the protein active site

The crystal models of Notum and CKIP-1 proteins chosen here presented the corresponding ligands. For the 4UZQ protein model, the corresponding ligands were palmitoleic acid and 2-acetamido-2-deoxy-beta-d-glucopyranose. Palmitoleic acid has been validated as a Notum protein inhibitor, and palmitoleic acid has been used in several studies to search for the corresponding inhibitor. Thus, the active site was determined using the receptor residues within the 4UZQ palmitoleic acid range and expanded to 9 Å using the ligand expansion method. For the 3AA1 protein model, the identified ligand was 2—(n-morphorino)—ethanesulfonic acid. The receptor residues in this range were chosen to determine the active site, and the ligand expanded to 9 Å.

### Preparation of small molecules

The small-molecule structure file of the SBT component with oral bioavailability (OB) ≥ 30% and drug resistance (DL) ≥ 0.18 was downloaded from the Traditional Chinese Medicine Systems Pharmacology Database and Analysis Platform (TCMSP). The three-dimensional structure of the small molecule was then downloaded from the TCMSP and loaded onto the DS docking software. Hydrogenation and energy optimization were performed using the CHARMM force field, and ligands were prepared and preserved for molecular docking as candidate small molecules.

### Molecular docking parameters

All molecular docking and screening in this study were performed using the CDOCKER function under the Dock Ligands module in DS. The target protein binding cavity’s x, y, and z coordinate positions were 13.173522, 38.263435, and 29.118304 for 4UZQ and 20.999862, 4.295655, and −4.878816 for 3AA1, respectively. The post-cluster radius was adjusted to 0.5, and default settings were used for the remaining parameters to ensure diversity of docked conformations.

### ADMT property prediction

SBT small molecules were initially screened based on the CDOCKER ENERGY score of molecular docking. Only the top five small molecules were considered in the subsequent analyses. The ADMET module in DS was then used to predict the activity of these small-molecule compounds, including water solubility, blood–brain barrier retention, CYP2D6 binding, liver toxicity, intestinal uptake, and plasma protein binding.

### Statistical analysis

Statistical analysis was performed by GraphPad Prism 9.0 software (San Diego, CA, United States). Continuous normally distributed data are presented as mean ± S.D., and differences between corresponding groups were analyzed using the Bonferroni’s or Dunnett’s *post hoc* test and one-way ANOVA. *p* < 0.05 was considered statistically significant.

## Results

### Micro-CT analysis of the femur

**FIGURE 1:**
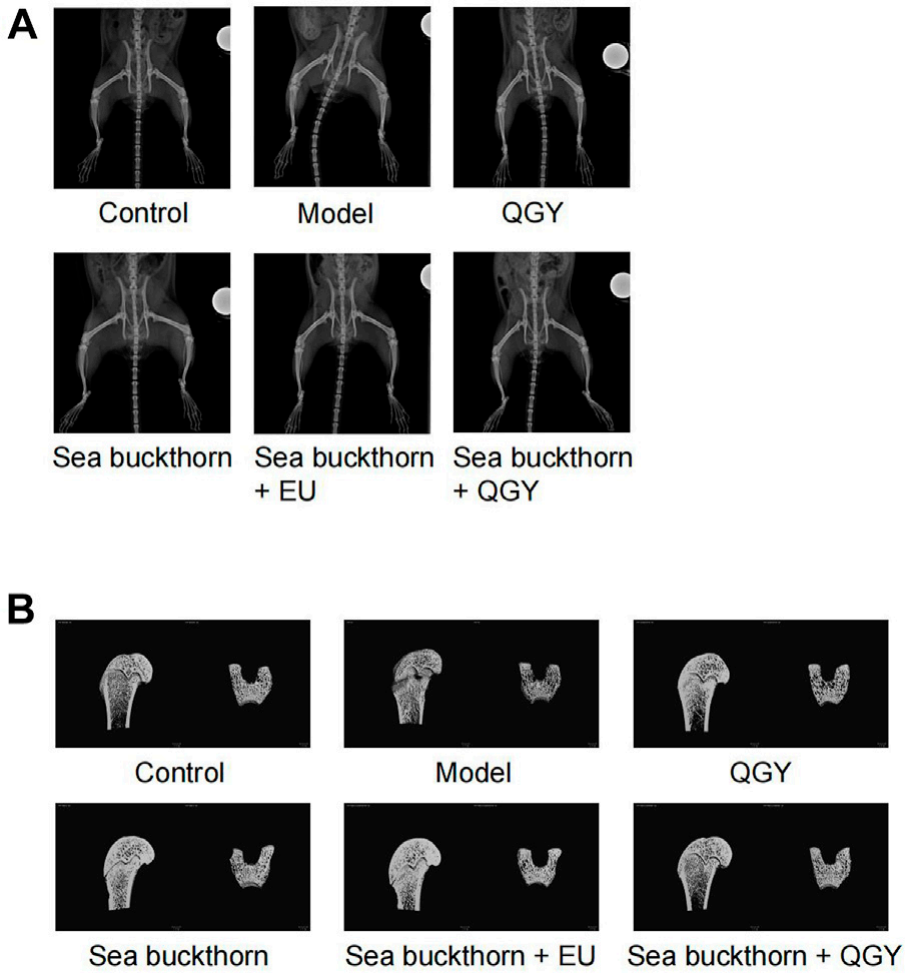
X-ray images of the rat hip and 3D images of the region of interest of the femur. **(A)** X-ray images showing the rat hip. **(B)** 3D images of the region of interest of the femur.

PMOP was established in a rat model to understand the effects of SBT and QGY. After treatment, the rats were scanned radiographically from the hip to the lower limb (Figure 1A), and the region of interest on the femur was reconstructed in three dimensions (Figure 1B).

**FIGURE 2:**
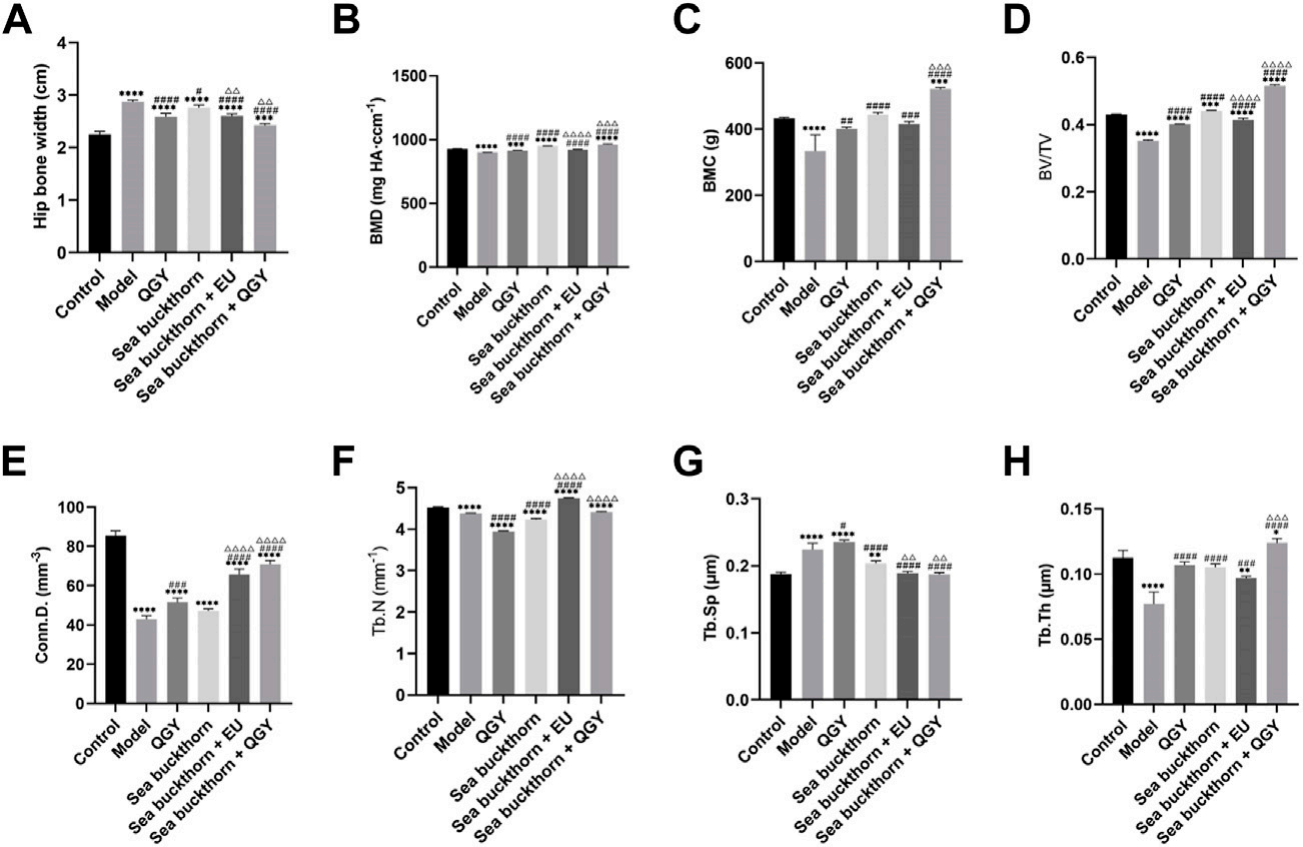
Analytical results of micro-CT for bone morphometry. **(A–H)** Results of the hip bone width, BMD, BMC, BV/TV, Conn.D. Tb.N, Tb.Sp, and Tb.Th. *, **, ***, and **** show *p* < 0.05, *p* < 0.01, *p* < 0.001, and *p* < 0.0001, respectively, in comparison with the normal control group; #, ##, ###, and #### show *p* < 0.05, *p* < 0.01, *p* < 0.001, and *p* < 0.0001, respectively, in comparison with the PMOP model group; △, △△, △△△, and △△△△ show *p* < 0.05, *p* < 0.01, *p* < 0.001, and *p* < 0.0001, respectively, in comparison with the QGY group.

Compared to the human hip bone, X-ray scanning revealed that the rat hip bone was long and curved. Thus, the area with the most significant curvature was selected for width measurement. This study found that compared with the control, the hip in the model group was significantly wider (*p* < 0.001), whereas the hip in all drug groups was significantly narrower (*p* < 0.001) than that in the model group. Meanwhile, the hip reduction was most significant in the SBT + QGY group. The effect was comparable for the QGY and SBT + EU groups. The SBT group displayed the least change (Figure 2A). The effect of SBT alone was inferior to the combination therapy groups, indicating that SBT enhanced the efficacy of QGY.

Three-dimensional reconstruction of the region of interest to the femoral head in rats revealed a significant decrease in BMD, BMC, BV/TV, Conn.D. Tb.N, and a significant increase in Tb.Sp in the model group, suggesting a successful PMOP induction. Compared to the model group, BMD, BMC, BV/TV, Conn.D, Tb.N, and Tb.Th in the drug groups showed almost a significant increase and a decrease in Tb. Sp. These results showed that the preparations containing SBT exerted a better effect than those without SBT (Figures 2B–H).

### ELISA analysis of estrogen, PINP, and S-CTX

**FIGURE 3:**
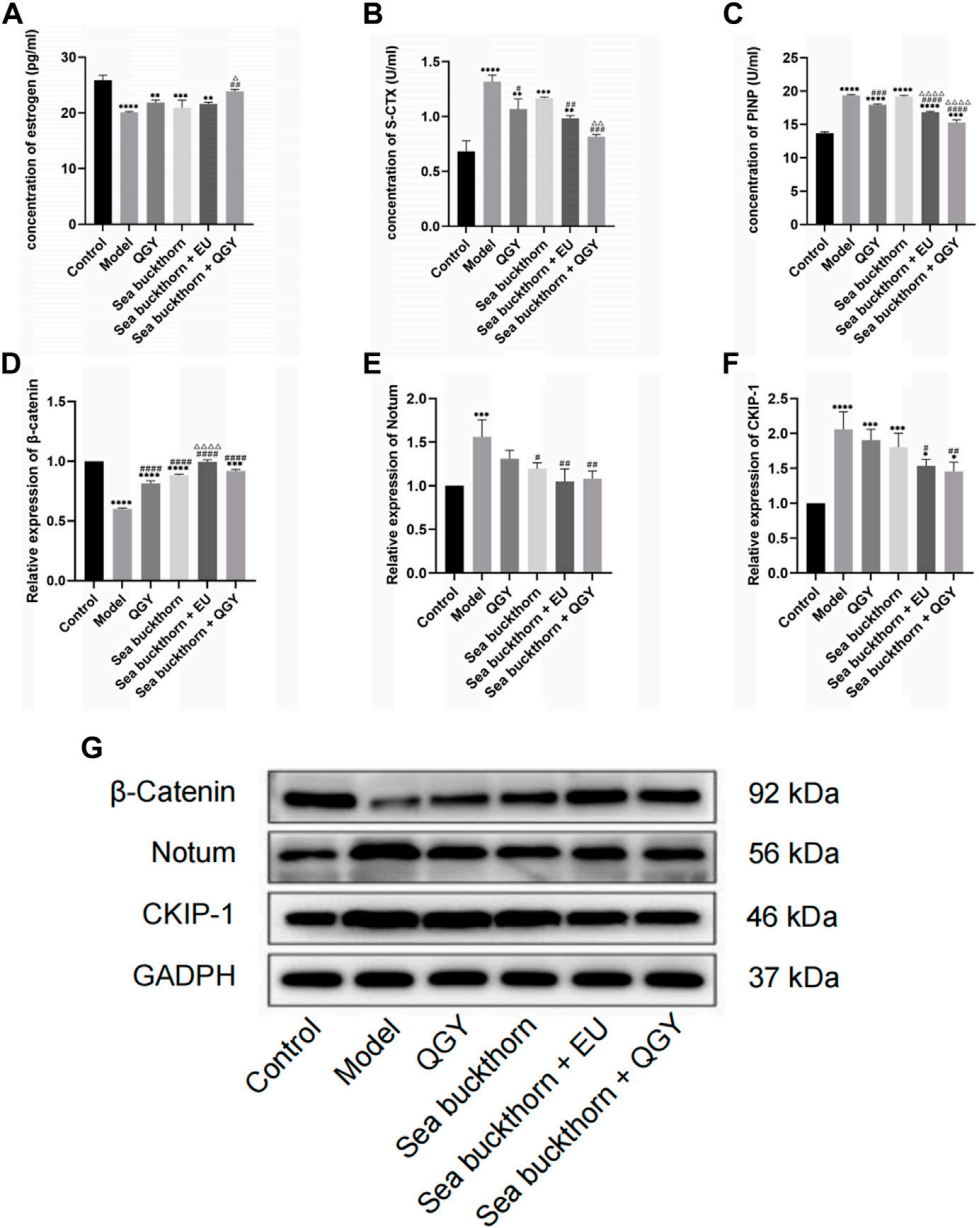
**(A–C)** The levels of estrogen, S-CTX and PINP. **(D–G)** the expression of β-catenin, Notum, and CKIP-1. *, **, ***, and ****, *p* < 0.05, *p* < 0.01, *p* < 0.001, and *p* < 0.0001, respectively, in comparison with the normal control group; #, ##, ###, and ####, *p* < 0.05, *p* < 0.01, *p* < 0.001, and *p* < 0.0001 respectively, in comparison with the PMOP model group; △, △△, △△△, and △△△△, *p* < 0.05, *p* < 0.01, *p* < 0.001, and *p* < 0.0001 respectively, in comparison with the QGY group.

Although the ELISA results showed that estrogen levels were significantly lower in the treatment group than those in the control group, they were still higher than those in the model group. The treatment can still effectively inhibit the decline of estrogen in rats. The findings also suggest that SBT + QGY was more effective in reducing estrogen levels than either medicine alone (Figure 3A).

The concentration of bone turnover markers PINP and S-CTX was significantly higher in the model group than that in the control group, suggesting high bone metabolism in rats, similar to PMOP. After treatment, the PINP concentration significantly decreased in the treatment groups except for the SBT group, which indicated that SBT alone had little effect on PINP. However, PINP reduction was higher in the SBT + QGY group than that in the QGY group (Figure 3C). This trend was also reflected in the expression of S-CTX (Figure 3B). This suggests that SBT enhances the efficacy of QGY, including inhibition of bone formation and bone absorption, but is more inclined to inhibit the latter.

### Western blot analysis of Notum, CKIP-1, and β-catenin

The effects of SBT on the expression of Notum and CKIP-1 and the regulation of the Wnt/β-catenin signaling pathway were assessed using the Western blot assay. Compared with the control group, SBT significantly decreased the expression of β-catenin but increased that of Notum and CKIP-1, all regulated *via* the Wnt/β-catenin signaling pathway. Inhibition of Notum and CKIP-1 expression is a common feature in PMOP.

This study found that all the drug groups suppressed the expression of Notum and CKIP-1 but promoted β-catenin expression (Figures 3D–G). Meanwhile, the effect of SBT + QGY was greater than that of either SBT or QGY alone, while the effect of SBT + EU was greater than that of either SBT or EU alone, further demonstrating that SBT inhibits osteoporosis. According to our results, SBT inhibits the expression of Notum and CKIP-1 by activating the Wnt/β-catenin signaling pathway. SBT further enhances the anti-osteoporotic properties of QGY.

### Molecular docking of SBT

**FIGURE 4:**
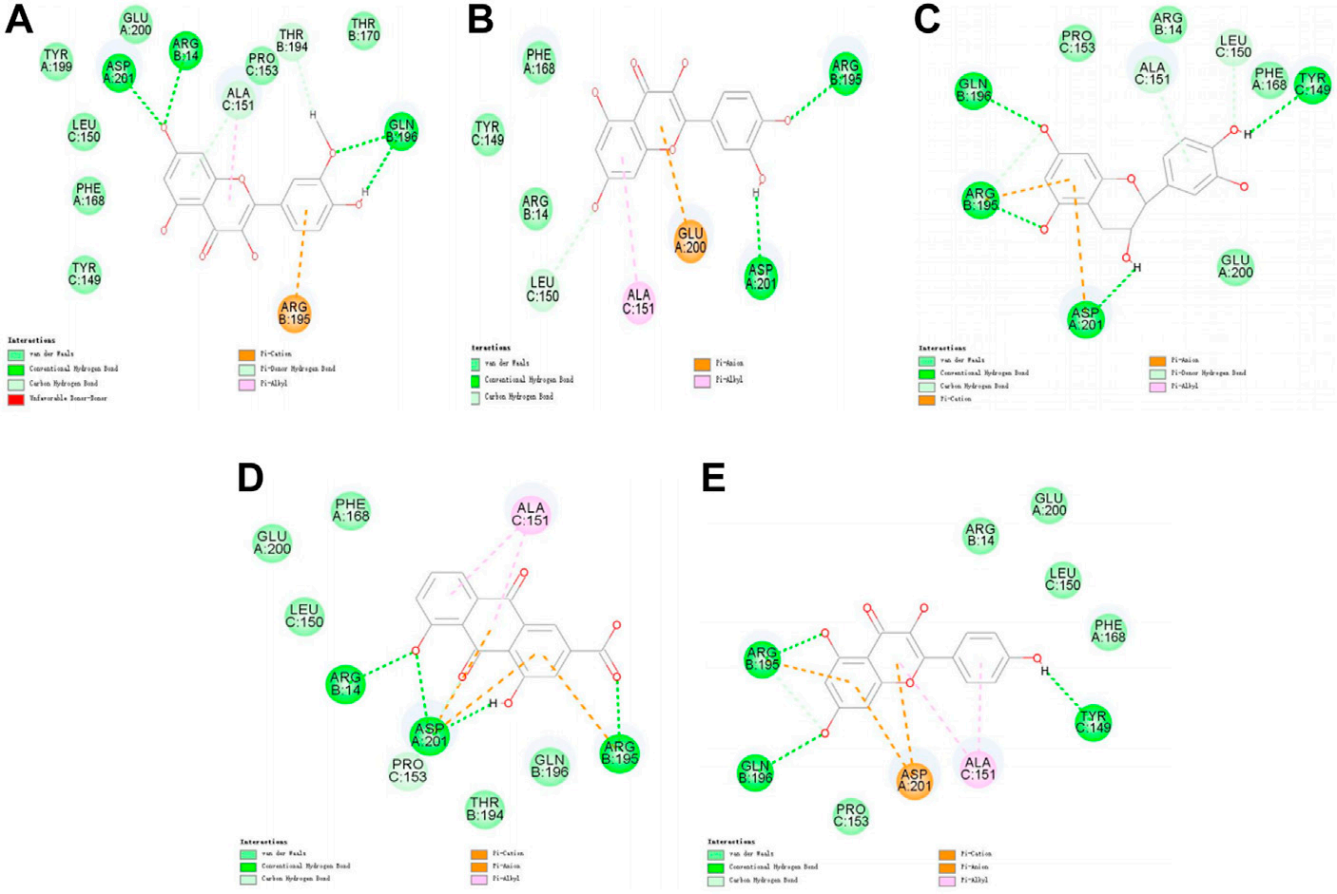
2D interaction map of CKIP-1 with selected small-molecule compounds. **(A)** Isorhamnetin; **(B)** quercetin; **(C)** ent-epicatechin; **(D)** rhein; **(E)** kaempferol.

**FIGURE 5:**
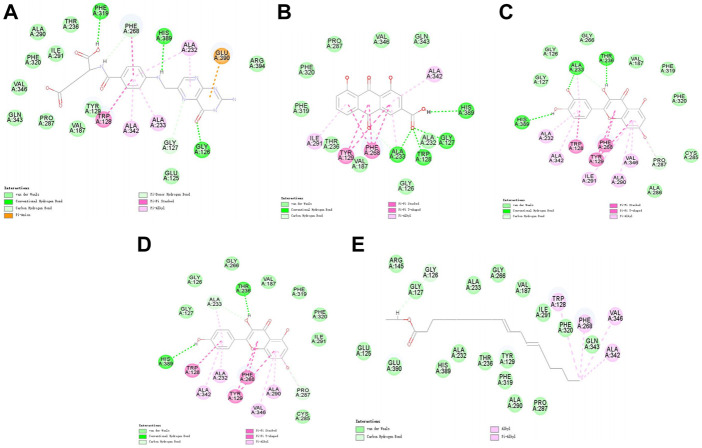
2D interaction map of Notum with selected small-molecule compounds. **(A)** Folic acid; **(B)** rhein; **(C)** quercetin; **(D)** kaempferol; **(E)** mandenol.

Molecular docking was performed on 33 prospective compounds with 4UZQ and 3AA1 receptors based on OB ≥ 30% and DL ≥ 0.18 (Table 1). The top five small molecules were selected for each protein score based on the CDOCKER ENERGY score (Figures 4, 5; Tables 2, 3).

**TABLE 1:** A total of 33 components of sea buckthorn after initial screening from the TCMSP database.

No.	MOL ID	Chemical name	OB	DL
1	MOL001004	Pelargonidin	37.99	0.21
2	MOL010211	14,15-Dimethyl-alpha-sitosterol	43.14	0.78
3	MOL010212	14-Methyl-alpha-sitosterol	43.49	0.78
4	MOL010227	Canthaxanthine	41.59	0.56
5	MOL010228	Carotenoid	40.76	0.55
6	MOL010230	ST5330591	48.08	0.84
7	MOL010232	cis-Lycopene	45.51	0.54
8	MOL010234	Delta-carotene	31.80	0.55
9	MOL010240	Ergosta-7-en-3-beta-ol	38.76	0.83
10	MOL010241	Ergostenol	35.41	0.71
11	MOL010247	(2R,6S,7aR)-2-[(1E,3E,5E,7E,9E,11E,13E,15E)-16-[(1R,4R)-4-hydroxy-2,6,6-trimethyl-1-cyclohex-2-enyl]-1,5,10,14-tetramethylhexadeca-1,3,5,7,9,11,13,15-octaenyl]-2,4,4,7a-tetramethyl-6,7-Dihydro-5H-benzofuran-6-ol	57.88	0.53
12	MOL010248	Gamma-carotene	30.98	0.55
13	MOL001979	LAN	42.12	0.75
14	MOL010267	LYC	32.57	0.51
15	MOL010283	ZINC03831331	47.60	0.65
16	MOL001420	ZINC04073977	38.00	0.76
17	MOL001494	Mandenol	42.00	0.19
18	MOL001510	24-Epicampesterol	37.58	0.71
19	MOL002268	Rhein	47.07	0.28
20	MOL002588	(3S,5R,10S,13R,14R,17R)-17-[(1R)-1,5-dimethyl-4-methylenehexyl]-4,4,10,13,14-pentamethyl-2,3,5,6,7,11,12,15,16,17-decahydro-1H-cyclopenta [a]phenanthren-3-ol	42.37	0.77
21	MOL002773	Beta-carotene	37.18	0.58
22	MOL000354	Isorhamnetin	49.60	0.31
23	MOL000358	Beta-sitosterol	36.91	0.75
24	MOL000359	Sitosterol	36.91	0.75
25	MOL000422	Kaempferol	41.88	0.24
26	MOL000433	Folic acid	68.96	0.71
27	MOL000449	Stigmasterol	43.83	0.76
28	MOL000492	(+)-Catechin	54.83	0.24
29	MOL005100	5,7-Dihydroxy-2-(3-hydroxy-4-methoxyphenyl) chroman-4-one	47.74	0.27
30	MOL006756	Schottenol	37.42	0.75
31	MOL000073	Ent-epicatechin	48.96	0.24
32	MOL000953	CLR	37.87	0.68
33	MOL000098	Quercetin	46.43	0.28

**TABLE 2:** Top five ingredients scored after docking 3AA1 molecules with 33 compounds.

No.	MOL ID	Chemical name	InCHI Key	CDOCKER ENERGY
1	MOL000433	Folic acid	OVBPIULPVIDEAO-LBPRGKRZSA-N	32.185
2	MOL002268	Rhein	FCDLCPWAQCPTKC-UHFFFAOYSA-N	31.788
3	MOL000098	Quercetin	REFJWTPEDVJJIY-UHFFFAOYSA-N	22.012
4	MOL000422	Kaempferol	IYRMWMYZSQPJKC-UHFFFAOYSA-N	21.700
5	MOL001494	Mandenol	FMMOOAYVCKXGMF-MURFETPASA-N	18.008

**TABLE 3:** Top five ingredients scored after docking 4UZQ molecules with 33 compounds.

No.	MOL ID	Chemical name	InCHI Key	CDOCKER ENERGY
1	MOL000433	Folic acid	OVBPIULPVIDEAO-LBPRGKRZSA-N	32.185
2	MOL002268	Rhein	FCDLCPWAQCPTKC-UHFFFAOYSA-N	31.788
3	MOL000098	Quercetin	REFJWTPEDVJJIY-UHFFFAOYSA-N	22.012
4	MOL000422	Kaempferol	IYRMWMYZSQPJKC-UHFFFAOYSA-N	21.700
5	MOL001494	Mandenol	FMMOOAYVCKXGMF-MURFETPASA-N	18.008

**FIGURE 6:**
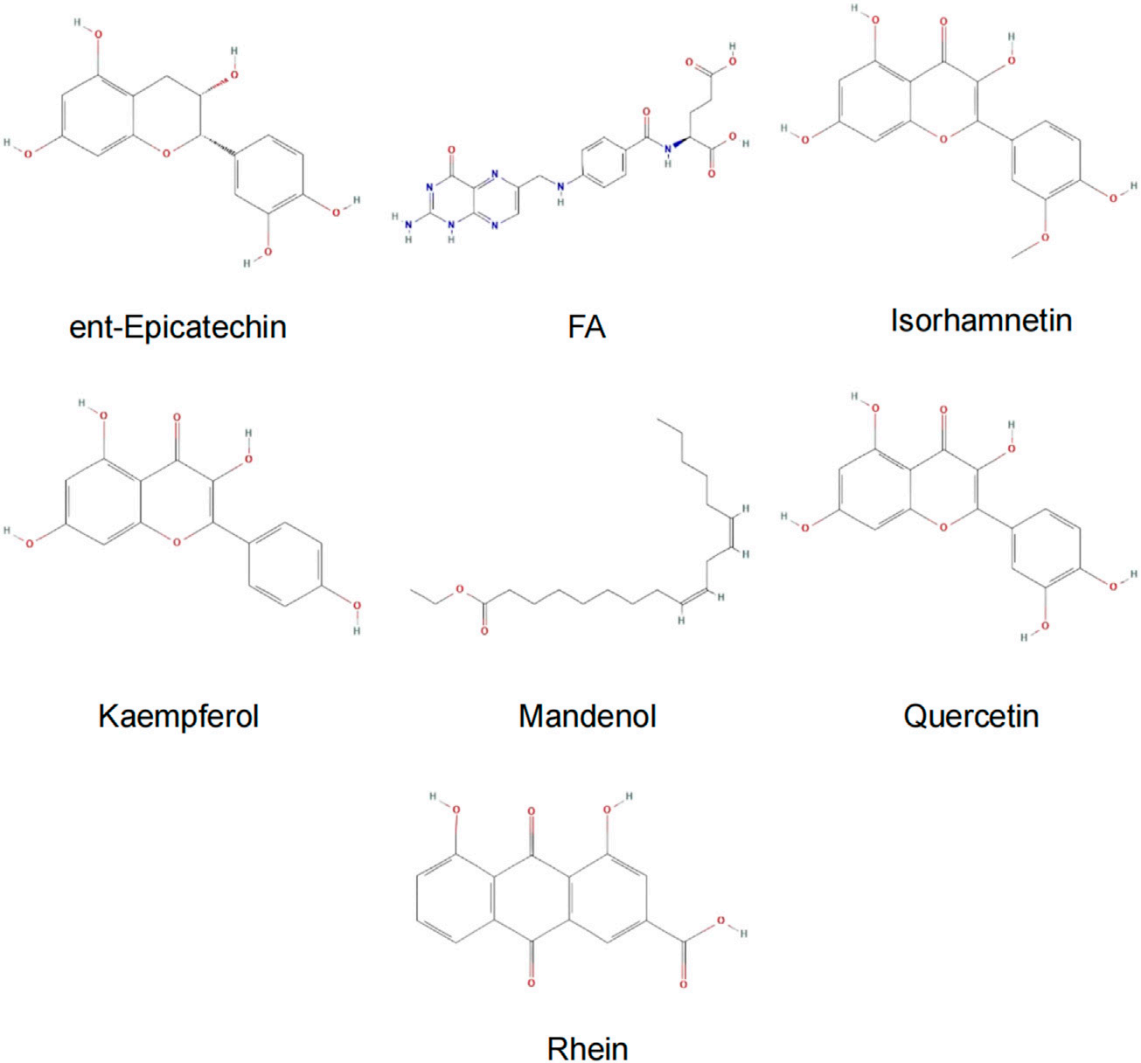
Active ingredients of sea buckthorn identified by virtual screening using molecular docking tools.

Among the 10 selected small molecules, three are repeated, namely, quercetin, rhein, and kaempferol (Figure 6). Folic acid and mandenol were unique to 4UZQ, while isorhamnetin and ent-epicatechin were unique to 3AA1. ADMET evaluation of the seven compounds showed that only folic acid, quercetin, and mandenol were absorbed into the human intestine, and folic acid and mandenol were less hepatotoxic. Mandenol binds to plasma protein more strongly than folic acid (Table 4).

**TABLE 4:** Results of ADMET screening.

No.	Chemical name	Molecular formula	Human intestinal absorptivity	Aqueous solubility (25°C)	Blood–brain barrier permeability	Cytochrome P450 2D6 inhibition	Hepatotoxicity	Plasma protein tuberculosis rate
1	Folic acid	C_19_H_19_N_7_O_6_	3	3	4	False	False	False
2	Rhein	C_15_H_8_O_6_	0	3	4	False	True	False
3	Quercetin	C_15_H_10_O_7_	1	3	4	False	True	False
4	Kaempferol	C_15_H_10_O_6_	0	3	3	False	True	False
5	Mandenol	C_20_H_36_O_2_	2	2	4	False	False	True
6	Isorhamnetin	C_16_H_12_O_7_	0	3	4	False	True	False
7	Ent-epicatechin	C_15_H_14_O_6_	0	3	4	False	True	False

Human intestinal absorptivity: 0–3 decreases in turn; water solubility (25°C): 0–6 increases in turn, and four is the best case; blood–brain barrier permeability: 0–5 decreases in turn; cytochrome P450 2D6 inhibition: true stands for inhibitory, and false for non-inhibitory; hepatotoxicity: true represents hepatotoxic, and false represents not hepatotoxic; plasma protein tuberculosis rate: true represents having high plasma protein binding, and false represents not having high plasma protein binding.

## Discussion

This study aimed to investigate the therapeutic effect of SBT on osteoporosis, the possible mechanism of action, active components in SBT, and whether it can improve the therapeutic effect of QGY. Previous clinical studies have shown that QGY is highly effective in treating PMOP (Shi, Liu, and Li, 2007; Shi et al., 2008, 2017; Xiao et al., 2019; Li et al., 2021; Mao et al., 2021; Hui et al., 2022). Studies have also shown that QGY promotes bone formation and inhibits bone resorption (Wu et al., 2008; Wang, Wu, and Shi, 2016; Yao et al., 2016; Liu et al., 2018; Yang et al., 2018; Huang, Shi, and Deng, 2019; Liang et al., 2019; Yuan et al., 2022). Thus, we studied the effects of SBT, QGY, SBT + QGY, and SBT + EU. The SBT + EU group was particularly important because EU is an important herb in QGY that strengthens bones. Multiple pharmacological studies have confirmed that EU inhibits bone resorption but promotes bone formation (MEHL, 2011; Tan et al., 2014; Zhang et al., 2014; Cheng et al., 2019; Qi et al., 2019; Jian et al., 2020; Zhao et al., 2020).

### Sea buckthorn enhances QGY efficacy on bone morphological parameters in PMOP rats

Micro-CT can demonstrate the three-dimensional structure of the bone more intuitively, which could relieve the clinical efficacy of therapies. In the study, micro-CT analysis showed that QGY, SBT, and EU, either alone or in any combination, increased bone morphometric parameters such as BMD, BMC, BV/TV, Conn.D, and Tb.Th. However, the Tb.N and Tb.Sp results were not anticipated. Intake of QGY and SBT decreased Tb.N, contrary to our expectations. Similarly, the increase in Tb.Sp was observed in the QGY group, which was also contrary to our expectation. But, SBT + QGY increased the Tb.N and decreased Tb.Sp, which is still in line with our expectations.

The bone metabolic index results revealed that S-CTX, an indicator of bone resorption, and PINP, a bone formation indicator, were both inhibited in the four treatment groups. The inhibition of S-CTX was higher in the SBT + QGY and SBT + EU groups than that in the QGY group, suggesting that SBT enhances the effect of QGY in inhibiting bone resorption and bone formation. We found that QGY significantly inhibited bone turnover, contrary to the previous studies. In the present study, the experiments were conducted for 6 weeks, whereas in other studies, the experiments were conducted for at least 2 months (Wang, Wu, and Shi, 2016; Yao et al., 2016; Huang, Shi, and Deng, 2019). Therefore, we speculate that QGY may inhibit bone turnover in the early stage of treatment. With regard to the degree of improvement in overall indicators, QGY effectively results in osteoporosis treatment, and SBT may improve its effect.

In clinical practice or other studies, the hip bone width has not been used in osteoporosis detection. In the present study, the hip bone was wider for rats in the model group than that in the control group, but the bone width decreased after treatment. This suggests that, unlike in humans, the hip bone may be an indicator of osteoporosis in rats. The hip bone was wider, but BMD, BMC, BV/TV, Conn.D, and Tb.Th were lower in the model rats, implying that degenerative bone changes may widen the hip joints of the rats.

### Sea buckthorn enhances QGY efficacy by inhibiting the expression of Notum and CKIP-1 and activating the Wnt/β-catenin signaling pathway

High estrogen levels and expression of β-catenin, Notum, and CKIP-1 were observed in all treatment groups, consistent with previous studies (Figures 3D–F). The inhibition of Notum and CKIP-1 was higher in the SBT + EU and SBT + QGY groups than that in the other groups. The activation of β-catenin was also higher in the SBT + EU and SBT + QGY groups than that in the other groups. Thus, we speculate that SBT activates the Wnt/β-catenin signaling pathway by inhibiting Notum and CKIP-1 expression.

The Wnt/β-catenin signaling pathway regulates bone metabolic homeostasis and participates in skeletal growth, development, repair, and remodeling. Studies show that β-catenin promotes the proliferation and differentiation of osteoblasts and inhibits osteoclasts during bone formation *via* the Wnt signaling pathway (A.Maupin, J.Droscha, and O.Williams, 2013). β-Catenin regulates bone resorption and modulates bone formation, thus impacting bone mass. β-Catenin also regulates osteoblast and osteoclast formation during osteoblast differentiation (Chen and Long, 2013).

Estrogen is a steroid hormone that regulates bone tissue metabolism by modulating the physiological activity of osteoblasts and osteoclasts (Zhao et al., 2007; Pereira et al., 2015). Estrogen binds to the estrogen receptor (ER) on the cell membrane and plays a corresponding biological role. On the one hand, estrogen regulates osteoclast activity *via* the RANKL/RANK pathway and osteoblast activity *via* the ERK1/2 pathway. On the other hand, it promotes the differentiation of bone marrow mesenchymal stem cells into osteoblasts by activating the Wnt signaling pathway (Gao et al., 2013). Therefore, based on the increase in estrogen levels in all four drug groups, we hypothesized that SBT and QGY activate the Wnt/β-catenin signaling pathway by increasing the production of estrogen and promoting bone formation *via* the RANKL/RANK/OPG axis. A recent study in rats has revealed that blocking the effects of estrogen on the hypothalamus increases the total bone mass by 500% and enhances the bone mechanical strength and mineral density throughout old age (Herber et al., 2019). In blood, estrogen promotes bone growth, but in the hypothalamus, it exerts an opposite effect. This study also showed us a new direction of anti-osteoporosis research.

Our research revealed that estrogen levels were significantly low, while CKIP-1 and Notum expression were significantly high in rats with PMOP. Further studies involving an estrogen production inhibition test are required to confirm the relationship between estrogen and CKIP-1, as well as Notum.

### Molecular docking screening identified seven potential active components that potentially affect CKIP-1 and Notum

Molecular docking identified seven small molecules in SBT, namely, folic acid, rhein, quercetin, mandenol, kaempferol, isosalmontin, and ent-epicatechin that potentially affect CKIP-1 and Notum expression. Quercetin, kaempferol, and isorhamnetin are members of the flavonoid family, specifically known for their antioxidant, wound healing, lipid regulation, antihypertensive, and immunomodulatory properties (Gupta et al., 2006; Pang et al., 2008, 2008; Pandurangan, Bose and Banerji, 2011; Hou et al., 2017, 2017). Folic acid is an important nutrient that participates in the metabolism of genetic materials and proteins, affecting animal reproductive performance and secretion of bile in the pancreas (Savini et al., 2019; Sato, 2020). Mandenol is a fatty acid that regulates blood glucose and protects the retina (Bouras et al., 2017; Gao et al., 2017). Ent-epicatechin is a polyphenol with antioxidant properties that protect the liver (Maheshwari et al., 2011; Guo et al., 2017). In addition, rhein possesses antitumor, antiviral, anti-inflammatory, and antifibrotic properties (Xu et al., 2016; Ge et al., 2017; Tang et al., 2017; Wang et al., 2018). These seven small molecules, however, are only the calculated results of molecular docking, and whether they promote or inhibit CKIP-1 and Notum expression remains to be explored further.

According to the ADMT property prediction results, only folic acid, quercetin, and mandenol are absorbed in the human intestine. Still, quercetin is hepatotoxic, and mandenol has a higher tuberculosis rate of plasma proteins than folic acid (Table 4). Therefore, the results of the present study show that mandenol has a very high clinical application potential. But these results were generated *in silico*. Thus, further experiments are needed to validate them. Regarding limitations, the findings of this study were not validated using experimental studies.

## Conclusion

SBT improves bone morphometric performance in PMOP rats by inhibiting CKIP-1 and Notum expression, increasing estrogen levels, and activating the Wnt/β-catenin signaling pathway. Furthermore, SBT enhances the properties of QGY. Folic acid, rhein, quercetin, kaempferol, mandenol, isorhamnetin, and ent-epicatechin are the most likely active ingredients in SBT. These results provide insight into the pharmacological mechanisms of SBT in treating osteoporosis.

## Data Availability

The original contributions presented in the study are included in the article/Supplementary Material; further inquiries can be directed to the corresponding authors.
